# Exercise counselling and referral in cancer care: an international scoping survey of health care practitioners’ knowledge, practices, barriers, and facilitators

**DOI:** 10.1007/s00520-022-07342-6

**Published:** 2022-09-29

**Authors:** Imogen Ramsey, Alexandre Chan, Andreas Charalambous, Yin Ting Cheung, H. S. Darling, Lawson Eng, Lisa Grech, Nicolas H. Hart, Deborah Kirk, Sandra A. Mitchell, Dagmara Poprawski, Elke Rammant, Margaret I. Fitch, Raymond J. Chan

**Affiliations:** 1 Rosemary Bryant AO Research Centre, Clinical and Health Sciences, University of South Australia, Adelaide, SA Australia; 2 School of Pharmacy & Pharmaceutical Sciences, Department of Clinical Pharmacy Practice, University of California, Irvine, CA USA; 3 Department of Nursing, Cyprus University of Technology, Limassol, Cyprus; 4 Department of Nursing, University of Turku, Turku, Finland; 5 School of Pharmacy, Faculty of Medicine, The Chinese University of Hong Kong, Hong Kong SAR, China; 6 Department of Medical Oncology, Command Hospital Air Force, Bangalore, India; 7 Division of Medical Oncology and Hematology, Department of Medicine, Princess Margaret Cancer Centre/University Health Network, University of Toronto, Toronto, Canada; 8 School of Clinical Sciences, Faculty of Medicine, Nursing and Health Sciences, Medicine Monash Health, Monash University, Melbourne, Australia; 9 Melbourne School of Psychological Sciences, University of Melbourne, Melbourne, Australia; 10 Health Sciences, Swinburne University, Melbourne, Australia; 11 Department of Cancer Experiences Research, Peter MacCallum Cancer Centre, Melbourne, Australia; 12 Caring Futures Institute, College of Nursing and Health Sciences, Flinders University, Bedford Park, SA Australia; 13 Exercise Medicine Research Institute, Edith Cowan University, Joondalup, WA Australia; 14 School of Nursing, Queensland University of Technology, Kelvin Grove, QLD Australia; 15 Institute for Health Research, University of Notre Dame Australia, Fremantle, WA Australia; 16 School of Nursing and Midwifery, Edith Cowan University, Joondalup, WA Australia; 17 Division of Cancer Control and Population Sciences, National Cancer Institute, Bethesda, MD USA; 18 Department of Oncology, King Faisal Specialist Hospital & Research Centre, Riyadh, Kingdom of Saudi Arabia; 19 College of Medicine and Public Health, Flinders University, Bedford Park, South Australia Australia; 20 Department of Human Structure and Repair, Ghent University, Ghent, Belgium; 21 Bloomberg Faculty of Nursing, University of Toronto, Toronto, ON Canada

**Keywords:** Exercise, Cancer survivors, Education, Guidelines, Physical activity, Knowledge translation

## Abstract

**Purpose:**

Evidence supports the role of prescribed exercise for cancer survivors, yet few are advised to exercise by a healthcare practitioner (HCP). We sought to investigate the gap between HCPs’ knowledge and practice from an international perspective.

**Methods:**

An online questionnaire was administered to HCPs working in cancer care between February 2020 and February 2021. The questionnaire assessed knowledge, beliefs, and practices regarding exercise counselling and referral of cancer survivors to exercise programs.

**Results:**

The questionnaire was completed by 375 participants classified as medical practitioners (42%), nurses (28%), exercise specialists (14%), and non-exercise allied health practitioners (16%). Between 35 and 50% of participants self-reported poor knowledge of when, how, and which cancer survivors to refer to exercise programs or professionals, and how to counsel based on exercise guidelines. Commonly reported barriers to exercise counselling were safety concerns, time constraints, cancer survivors being told to rest by friends and family, and not knowing how to screen people for suitability to exercise (40–48%). Multivariable logistic regression models including age, gender, practitioner group, leisure-time physical activity, and recall of guidelines found significant effects for providing specific exercise advice (*χ*^2^(7) = 117.31, *p* < .001), discussing the role of exercise in symptom management (*χ*^2^(7) = 65.13, *p* < .001) and cancer outcomes (χ^2^(7) = 58.69, *p* < .001), and referring cancer survivors to an exercise program or specialist (*χ*^2^(7) = 72.76, *p* < .001).

**Conclusion:**

Additional education and practical support are needed to equip HCPs to provide cancer survivors with exercise guidelines, resources, and referrals to exercise specialists.

**Supplementary Information:**

The online version contains supplementary material available at 10.1007/s00520-022-07342-6.

## Background

Exercise uses structured and repetitive movement to achieve a targeted physiologic response [1] and is well-established as a safe and beneficial therapy for cancer survivors to counteract adverse cancer- and treatment-related side effects while improving health-related quality of life [2]. Epidemiological studies demonstrate associations between physical activity (PA; which refers to any movement produced by skeletal muscles that requires energy expenditure) and reduced risks of cancer recurrence, progression or new primaries, cardiovascular disease-related morbidity, and cancer-specific and all-cause mortality for some cancer survivors post-diagnosis [3–6]. Strong evidence demonstrates that exercise reduces prevalent and debilitating issues including cancer-related fatigue and psychological distress, while increasing physical function (i.e. body composition, muscle strength, aerobic fitness, and functional capacity) and health-related quality of life [7–10]. This evidence prompted major international organisations to endorse exercise as an essential part of cancer care [11]. Original guidelines for cancer survivors resembled those for the general population, encouraging 75 to 150 min of moderate-to-vigorous-intensity PA and two or more sessions of resistance training per week to achieve meaningful health benefits [11, 12]. More specific and structured exercise recommendations for most cancer survivors [13, 14], including those with advanced cancer and bone metastases [15], have been recently revised or developed.

Despite the well-documented benefits of PA and structured exercise [3–10], and dissemination of clinical exercise guidelines [13–15], approaches translating this evidence into practice are lacking [16]. Estimates of cancer survivors who meet PA guidelines range from about 10 to 30% [17]. Research has identified that barriers to exercise include cancer-related symptoms, low mood, time limitations, low exercise literacy, low awareness of exercise benefits, and lack of advice or referral from a health care practitioner (HCP) [18–21]. Other influential factors include sociodemographic and clinical variables, obesity, smoking, alcohol consumption, attitudes towards exercise, self-efficacy, and intentions [22–24]. HCPs are instrumental in facilitating health behaviour change in cancer survivors as trusted agents of information, and are well-placed to discuss and recommend exercise, and refer to exercise specialists [16].

Studies conducted largely in high-income countries highlight various barriers to translating evidence to practice. Cancer survivors receive advice from their HCPs to begin, resume, continue, or advance an exercise program [25], but studies suggest that many cancer survivors (30–70%) are not advised to exercise by a HCP [26–29]. Identified barriers to HCPs implementing exercise guidelines include perceiving some cancer survivors as unsuitable relative to current guidelines, safety concerns, limited time, perceived scope of practice, insufficient knowledge, unwillingness of cancer survivors to adopt recommendations, and anticipated failure in altering individuals’ behaviour [26, 27, 30–34]. Studies have reported that the greatest barrier to oncology HCPs discussing exercise with cancer survivors was limited knowledge about the existence and implementation of clinical exercise guidelines, despite appreciating the benefits of exercise [35, 36]. Specifically, most were either not aware of exercise guidelines in cancer, or had poor knowledge on when, how, and which cancer survivors to refer to exercise programs or exercise specialists [32], highlighting a knowledge-to-action (KTA) gap. Additionally, evidence suggests that HCPs who are physically active themselves are more likely to provide exercise counselling [37].

Building on previous research using the KTA framework [32, 38], the Multinational Association for Supportive Care in Cancer (MASCC) Survivorship Study Group conducted the first international study to investigate HCPs’ knowledge, beliefs, practices, and barriers and facilitators regarding exercise counselling and referral for cancer survivors. Secondary objectives were to explore differences across practitioner groups (i.e. medical practitioners, nurses, exercise specialists, non-exercise allied health practitioners) and factors influencing clinical practice. Sampling HCPs internationally will provide novel insights for knowledge exchange and innovations to address this KTA gap.

## Methods

### Study design

A cross-sectional online survey was conducted between February 2020 and February 2021. Human research ethics approval was obtained from the University of South Australia (ID: 202,287) and Edith Cowan University (ID: 2019–00,620-WALKER) Human Research Ethics Committees.

### Sample and recruitment

Participants were recruited using convenience and snowball sampling. Active recruitment efforts commenced in February 2020 and concluded in February 2021 with a 6-month break (April to September) due to the COVID-19 pandemic. To capture a broad range of HCPs involved in cancer care, eligible participants were practising HCPs who reported spending at least 10% of their work time providing direct clinical care to cancer survivors. To reach a multidisciplinary and global audience, the study was promoted online (i.e. via email, social media, newsletters, and webpages) by international oncology organisations, HCP societies, and members of the research team (see Supplement 1). They distributed a survey link and a reminder approximately 4 weeks after the initial invitation. Information about the nature and purpose of the study was provided at the beginning of the survey and participants were informed that completion of the survey implied their consent to participate. Participants were required to indicate that they fulfilled the inclusion criteria before they could commence the survey. All responses were anonymous.

### Measures

The Clinicians’ Perspectives on Exercise in Patients with Cancer (CliPEC) questionnaire was used [39]. The questionnaire was developed based on knowledge translation theory and validated using the Theoretical Domains Framework (TDF), which identifies potential targets for HCP behaviour change related to evidence-based practice [39]. CliPEC items were mapped to TDF domains, and the questionnaire demonstrated good internal consistency and construct validity [39]. The CliPEC questionnaire assesses HCPs’: (1) demographics (9 multiple choice and free-text items); (2) knowledge of exercise guidelines (5 items rated on a scale from 1 = ‘no knowledge’ to 7 = ‘very knowledgeable’ and 2 free-text items assessing their recall of exercise guidelines for cancer survivors); (3) attitudes regarding the benefits of exercise for cancer survivors (8 items rated on a scale from 1 = ‘strongly disagree’ to 7 = ‘strongly agree’); (4) current practices in exercise promotion, advice, and referral (6 items rated on a scale from 1 = ‘very unlikely’ to 7 = ‘very likely’); and (5) barriers and facilitators to exercise counselling and program referral (13 barriers rated on a scale from 1 = ‘strongly disagree’ to 7 = ‘strongly agree’ and 10 facilitators rated on a scale from 1 = ‘not at all helpful’ to 7 = ‘very helpful’) [39]. Minor modifications were made to the wording of some items to improve their relevance to an international audience for this study.

Weekly participation in self-reported leisure-time PA of HCPs in this study was assessed using the validated Godin-Shephard Leisure-Time Exercise Questionnaire [40]. Consistent with national and international PA and exercise guidelines, participants were classified according to whether or not they participated in ≥ 150 min of moderate-intensity exercise, ≥ 75 min of vigorous-intensity exercise, or a combination of the two per week [13–15].

### Statistical analyses

Descriptive analyses were conducted to examine measures of central tendency, dispersion and shape of the distributions for continuous variables, and percentage distributions for categorical variables. Based on previous research and the skewed distribution of responses, scores for knowledge and current practice items were dichotomised to ‘poor’ or ‘unlikely’ (1–4) and ‘good’ or ‘likely’ (5–7) [32, 39]. Chi-square tests were performed to explore differences based on practitioner group and whether participants reported meeting recommended weekly participation in leisure-time PA according to widely accepted guidelines. Predictors of HCPs’ current practices were examined using multivariable logistic regression. Variables were entered using purposeful selection, informed by published literature and univariate analyses. Factors included in the final models were age, gender, practitioner discipline, knowledge of oncology exercise guidelines (as indicated by accurate recall), and self-reported leisure-time PA. Tests were two-tailed and considered significant if *p* < .05. Analyses were conducted using IBM SPSS Statistics.

## Results

A total of 375 participants completed the survey. Sample characteristics are presented in Table 1. The majority of respondents were female (77%), the median age was 43 years, and the median duration of practice was 15 years. Around 40% of participants were medical practitioners, 28% were nurses, 14% were exercise specialists, and 29% were non-exercise allied health practitioners. Exercise specialists included kinesiologists, exercise physiologists, physiotherapists, and other qualified exercise professionals. The complete list of participants’ professional roles and areas of clinical practice is provided in Table 1. Participants practised in the Asia–Pacific region (*n* = 161, 43%), North America (*n* = 49, 13%), Western Europe (*n* = 102, 27%), Eastern Europe (*n* = 45, 12%), Latin America (*n* = 9, 2%), and Africa (*n* = 6, 2%), representing 45 countries in total. Most countries where participants practised were classified as high income (89%). Participants most reported that they provided care to cancer survivors from all disease sites (41%), followed by breast (29%), genitourinary (22%), or gastrointestinal (19%) cancers. More than half reported participating in ≥ 150 min of moderate-intensity or ≥ 75 min of vigorous-intensity leisure-time PA per week, and this did not differ across practitioner groups.

**Table 1:** Participant characteristics (*n* = 373)

Characteristic	*n*	%
Gender (*n* = 373)		
Female	86	22.9
Male	287	76.5
Years of age (*n* = 368)		
Median	43	
Range	22 to 72	
Years in practice (*n* = 368)		
Median	15	
Range	1 to 50	
Primary clinical role categories (*n* = 371)		
Medical practitioner	155	
Physician	148	39.5
Paediatrician	4	1.1
Radiation oncologist	1	0.3
Registrar	2	0.5
Nurse	105	
Advanced practice nurse	1	0.3
Clinical nurse specialist	42	11.2
Nurse consultant	11	2.9
Nurse practitioner	10	2.7
Registered nurse	41	10.9
Exercise specialist	53	
Exercise physiologist	4	1.1
Physiotherapist	48	12.8
Cancer exercise specialist	1	0.3
Allied health practitioners (non-exercise)	58	
Dietician	7	1.9
Medical physicist	2	0.5
Occupational therapist	4	1.1
Pharmacist	2	0.5
Psychologist	2	0.5
Radiation therapist	38	10.1
Social worker	1	0.3
Speech pathologist	2	0.5
Primary practice setting		
Community, government or municipal hospital/tertiary care centre	184	49.1
Private practice	41	10.9
Academic institution	95	25.3
Comprehensive cancer centre	75	20.0
Hospice/end-of-life care	11	2.9
Government organisation	9	2.4
Non-government organisation	17	4.5
Other	6	1.6
Types of cancer treated on regular basis		
All cancers	152	40.5
Breast	107	28.5
Lung	72	19.2
Gastrointestinal	83	22.1
Head and neck	59	15.7
Gynaecological	64	17.1
Central nervous system	47	12.5
Melanoma and skin	33	8.8
Sarcoma	34	9.1
Haematological (leukaemia)	31	8.3
Haematological (lymphoma, myeloma)	42	11.2
Genitourinary	82	21.9
Main area(s) of clinical practice		
Radiation oncology	184	49.1
Medical oncology	156	41.6
Haematology/oncology	81	21.6
Symptom management and palliative care	65	17.3
Surgical oncology	58	15.5
Psycho-oncology	17	4.5
Rehabilitation	52	13.9
Other	34	9.1
Region (*n* = 372)		
Asia–Pacific	161	42.9
Western Europe	102	27.2
North America	49	13.1
Eastern Europe	45	12.0
Latin America	9	2.4
Africa	6	1.6
Country income classification (*n* = 372)		
High income	330	88.0
Upper middle income	28	7.5
Lower middle income	14	3.7
Exercise minutes per week (*n* = 338)		
≤ 150 moderate or ≤ 75 vigorous or an equivalent combination	152	40.5
≥ 150 moderate or ≥ 75 vigorous or an equivalent combination	186	49.6

### Knowledge of exercise guidelines

**Fig. 1:**
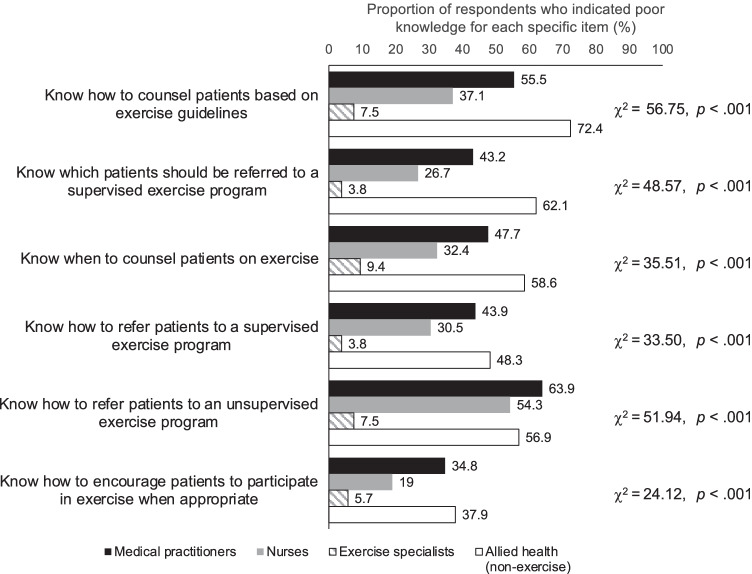
Participants’ self-reported knowledge of exercise counselling, by practitioner group

Between 35 and 50% of participants self-reported low levels of knowledge with respect to when, how, and which cancer survivors to refer to exercise programs or exercise specialists, and how to counsel based on exercise guidelines (Fig. 1). More than 90% of exercise specialists self-reported good knowledge in all domains. Around half of the medical practitioners, two-thirds of nurses, and one-third of non-exercise allied health practitioners reported knowing how and when to counsel cancer survivors based on exercise guidelines. A higher proportion reported knowing how to encourage cancer survivors to exercise when appropriate. Over half of medical practitioners self-reported good knowledge about which cancer survivors could be referred to a supervised exercise program and how to refer them. Self-reported knowledge in these areas was higher for nurses and lower for non-exercise allied health practitioners. However, only 22% of medical practitioners, 30% of nurses, 65% of exercise specialists, and 7% of non-exercise allied health practitioners accurately recalled how many minutes of aerobic exercise and how many sessions of resistance training are typically recommended for cancer survivors in international guidelines.

Compared with those who did not, participants who reported meeting PA guidelines themselves were more likely to self-report good knowledge about how to counsel cancer survivors based on exercise guidelines (*χ*^2^ = 5.22; *p* = .022), when to counsel about exercise (*χ*^2^ = 11.37; *p* = .001), which cancer survivors should be referred for exercise (*χ*^2^ = 6.56; *p* = .010), how to refer them to a supervised (*χ*^2^ = 7.19; *p* = .007) or unsupervised exercise program (*χ*^2^ = 5.29; *p* = .022), and how to encourage cancer survivors to participate in exercise (*χ*^2^ = 14.56; *p* < .001).

### Beliefs

Most participants disagreed that cancer survivors should avoid exercise when they feel fatigued (89%), that exercise will worsen cancer pain (86%), and that people with metastatic disease should not exercise (94%). Most agreed that moderate levels of exercise can decrease the risk of disease recurrence for some cancer types (73%) and that exercise counselling should be a part of the care they provide (92%). Exercise beliefs did not vary across practitioner groups.

### Practice patterns

Approximately two-thirds of respondents (68%) reported that exercise counselling was a routine component of the care they provide (68%). Those that reported providing these interventions were also more likely to report meeting PA guidelines themselves (*χ*^2^ = 8.72; *p* = .003). Of the cancer survivors that participants considered safe to exercise, most reported that they would be likely to advise them to keep active before (87%), during (93%), and after (94%) treatment, and to discuss the role of exercise in symptom management (83%). Fewer indicated they would be likely to discuss the role of exercise for cancer outcomes (65%), provide specific exercise guidelines or advice (56%), or refer cancer survivors to an exercise program or exercise specialist (61%).

Participants who reported meeting PA guidelines themselves were more likely to discuss the role of exercise in symptom management (*χ*^2^ = 11.32; *p* = .001) or cancer outcomes (*χ*^2^ = 8.19; *p* = .004), provide specific exercise guidelines or advice (*χ*^2^ = 11.23; *p* = .001), or refer cancer survivors to an exercise program (*χ*^2^ = 12.47; *p* < .001). Similarly, participants who accurately recalled exercise guidelines were more likely to discuss the role of exercise in symptom management (*χ*^2^ = 17.08; *p* < .001) or cancer outcomes (*χ*^2^ = 23.99; *p* < .001), provide specific exercise guidelines or advice (*χ*^2^ = 48.30; *p* < .001), or refer cancer survivors to an exercise program (*χ*^2^ = 33.62; *p* < .001). Practices did not differ significantly based on country income. Women were more likely than men to discuss the role of exercise in symptom management (*χ*^2^ = 5.17; *p* = .023) or refer cancer survivors to an exercise program (*χ*^2^ = 5.82; *p* = .016).

When participants were asked about who should be responsible for discussing exercise with cancer survivors, 94% nominated exercise specialists (physiotherapists, kinesiologists, and exercise specialists), followed by nurses (66%), physicians (65%), and occupational therapists (51%).

### Factors associated with practices

Logistic regression results are reported in Table 2. A model examining associations between age, practitioner discipline, knowledge of exercise guidelines, and self-reported leisure-time PA and the provision of specific exercise guidelines or advice was significant (*χ*^2^(7) = 117.31, *p* < .001) and explained 36.6% of the variance (Nagelkerke *R*^2^). Participants who were older, had better knowledge of exercise guidelines, and who met PA guidelines were significantly more likely to provide specific exercise guidelines or advice, and exercise specialists were also more likely to provide these interventions compared to medical practitioners.

**Table 2:** Factors associated with exercise counselling and referral practices, multivariable logistic regression (*n* = 367)

	Discuss the role of exercise in symptom management		Discuss the role of exercise for cancer outcomes		Provide specific exercise guidelines or advice		Refer to an exercise program	
	*OR (CI)*	*p*	*OR (CI)*	*p*	*OR (CI)*	*p*	*OR (CI)*	*p*
Age (years)	1.06 (1.03–1.10)	** < .001**	1.04 (1.02–1.07)	**.001**	1.06 (1.03–1.08)	** < .001**	1.02 (1.00–1.04)	.087
Gender								
Male (ref)								
Female	1.62 (0.82–3.18)	.164	1.17 (0.67–2.05)	.59	0.97 (0.53–1.75)	.923	1.53 (0.88–2.69)	.135
Practitioner group								
Medical (ref)								
Exercise	4.26 (0.89–20.32)	.069	3.56 (1.39–9.12	**.008**	31.85 (6.98–145.29)	** < .001**	13.74 (3.10–61.01)	**.001**
Allied health	0.40 (0.19–0.82)	**.012**	0.60 (0.31–1.14)	.121	0.90 (0.45–1.77)	.753	.894 (0.47–1.69)	.716
Nursing	1.46 (0.63–3.37)	.381	1.52 (0.84–2.76)	.17	1.77 (0.99–3.18)	.056	1.11 (0.55–1.69)	.894
Knowledge								
Poor (ref)								
Good	3.50 (1.28–9.58)	**.015**	2.62 (1.41–4.86)	**.002**	4.24 (2.25–8.02)	** < .001**	3.10 (1.68–5.73)	** < .001**
Meets exercise guidelines								
No (ref)								
Yes	2.42 (1.29–4.53)	**.006**	1.62 (1.01–2.59)	**.044**	1.73 (1.07–2.82)	**.044**	1.89 (1.19–3.00)	**.007**

Eight cases with missing data were excluded from analyses. *OR* odds ratio, *CI* confidence interval. Significant *p* values (> .05) are in bold

Models investigating the effects on discussing the role of exercise in symptom management and cancer outcomes were also significant (symptom management: *χ*^2^(7) = 65.13, *p* < .001; cancer outcomes: *χ*^2^(7) = 58.69, *p* < .001), explaining 26.9% and 20.4% of the variance, respectively. Participants who were older, had better knowledge of exercise guidelines, and met PA guidelines were more likely to have these discussions.

The model investigating the effects of referring cancer survivors to an exercise program or exercise specialist was significant (*χ*^2^(7) = 72.76, *p* < .001) explaining 24.4% of the variance. Participants who had better knowledge of exercise guidelines or met PA guidelines themselves were more likely to refer cancer survivors, and exercise specialists were more likely to than medical practitioners.

### Barriers and facilitators

**Fig. 2:**
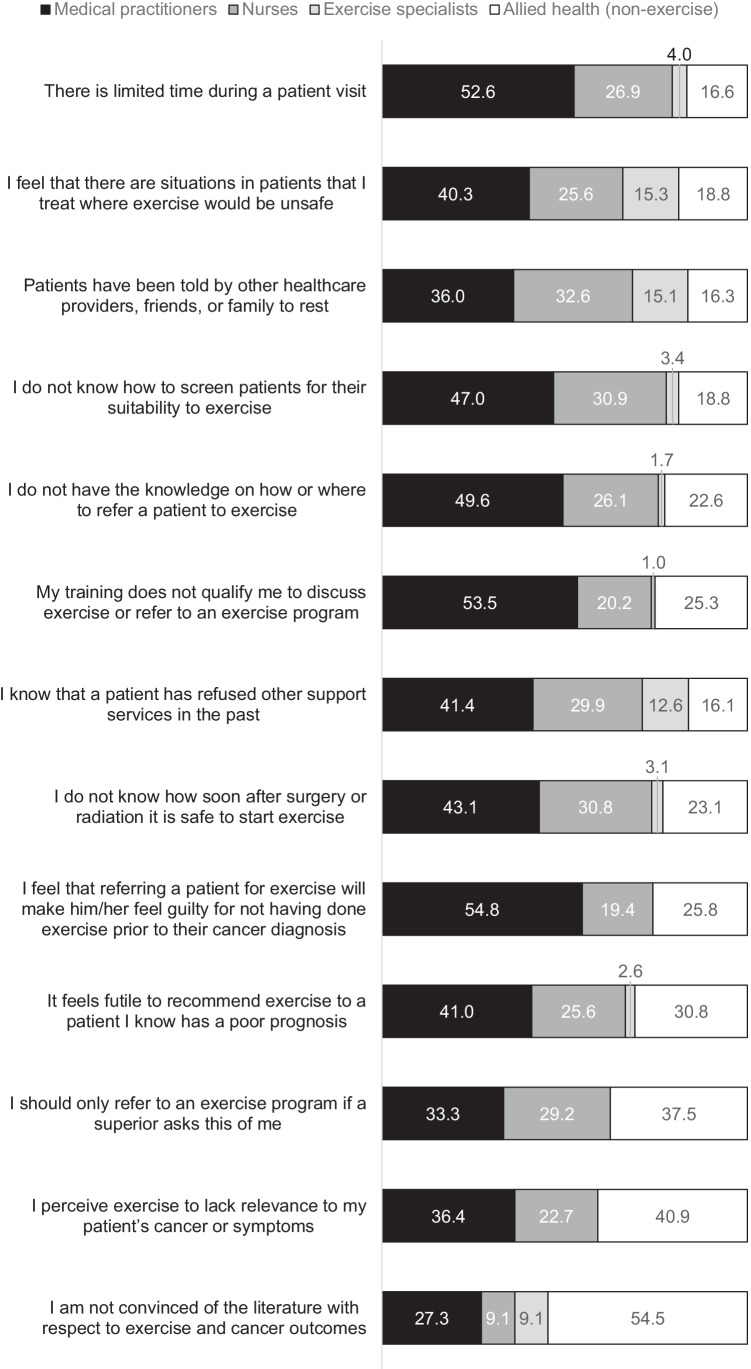

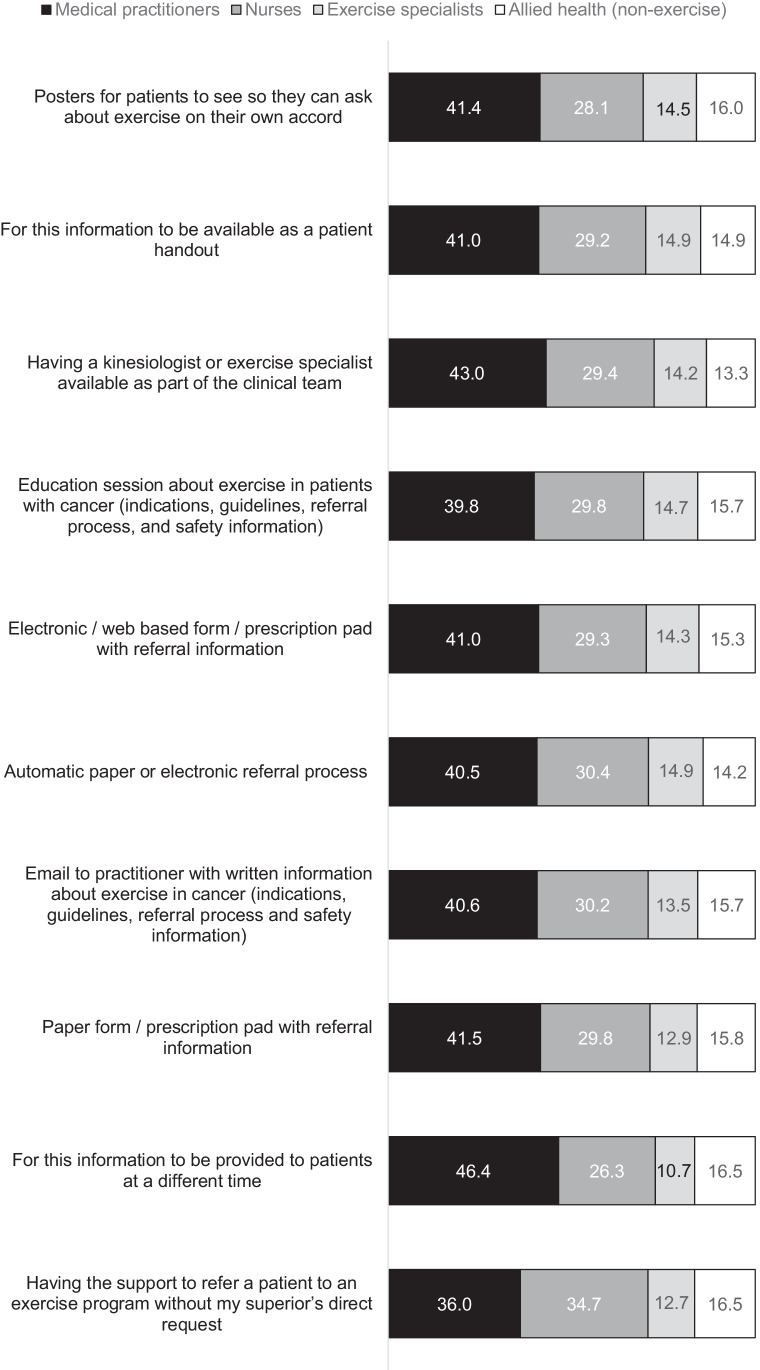
Barriers and facilitators to participants discussing exercise with cancer survivors, presented as the proportion of agreement by practitioner group

The top four barriers to participants discussing exercise were safety concerns (48%), limited time during visits (47%), cancer survivors being told by friends and family to rest (46%), and not knowing how to screen cancer survivors for their suitability and safety to exercise (40%). These were the top four barriers for medical practitioners, nurses, and non-exercise allied health practitioners (Fig. 2). When asked to specify their safety concerns, participants most frequently mentioned cancer survivors who have bone metastases and high risk of fractures, mobility or balance issues, acute treatment side effects, and cardiac comorbidities, and cancer survivors who are frail, elderly, or palliative. Other common additional barriers specified by participants were limited availability of or access to suitable programs, lack of funding or resourcing, cost to cancer survivors, and lack of knowledge.

All facilitators were endorsed by more than 60% of the participants. The top four facilitators for participants discussing exercise (Fig. 2) were posters (89%), handouts for cancer survivors (89%), practitioner education sessions (86%), and having a kinesiologist or exercise specialist as part of the clinical team (85%).

## Discussion

We surveyed international HCPs about their knowledge, beliefs, practices, barriers, and facilitators regarding providing exercise counselling to cancer survivors. Participants generally agreed that exercise counselling should be part of their routine care, although only two-thirds agreed that it was part of their routine care. A higher proportion of participants self-reported good knowledge about when and how to counsel cancer survivors based on exercise guidelines than accurately recalled recommended exercise guidelines. The most common barriers to exercise counselling reported by non-exercise HCPs were safety concerns, time constraints, cancer survivors being told to rest by family or friends, and not knowing how to screen cancer survivors for suitability to exercise. Predictors of exercise counselling and referral were knowledge of current guidelines, HCPs themselves meeting common PA guidelines, and older age.

Compared with previous studies, the proportion of participants who self-reported being knowledgeable about exercise counselling and referral was relatively high [32]. Nevertheless, 35–52% of the participants self-reported low levels of knowledge of when, how, and which cancer survivors to refer to exercise programs, and how to counsel them based on exercise guidelines (Table 2). This was lower than a study of Canadian HCPs, in which approximately 80% of participants self-reported poor knowledge across these domains [32]. However, only 22% of medical practitioners, 30% of nurses, 65% of exercise specialists, and 7% of non-exercise allied health practitioners in our study accurately recalled cancer exercise guidelines. Lack of knowledge about how to screen cancer survivors for suitability to exercise and where and how to refer cancer survivors to an exercise program were commonly cited barriers by non-exercise HCPs. These results suggest that despite being relatively high [32], knowledge remained a key gap. Given that knowledge is hypothesised to influence behaviour change and was a key predictor of participants’ current practices, increasing educational and engagement opportunities for HCPs may support the implementation of exercise guidelines in their routine practice [41].

The proportion of participants who reported that they advised cancer survivors to keep physically active before, during, and after treatment was higher than that reported in surveys of HCPs conducted in Germany and the USA [42, 43]. Studies investigating exercise guideline implementation in cancer have typically focused on factors that influence HCPs’ beliefs and practices regarding recommending PA or exercise. However, as argued by Santa Mina and colleagues [16], the strategy of simply making recommendations fails to capitalise on the opportunity present during a clinical interaction. In their pathway model of engaging people with cancer in PA and exercise, the authors propose that more targeted strategies are required to stimulate and support a greater proportion of cancer survivors to participate in exercise as a long-term adjuvant component of therapy [16]. This is supported by studies that have found improved participation in exercise and outcomes for cancer survivors when a recommendation was provided in addition to an outlet or resource, compared with a recommendation only [44, 45]. Our study participants were less likely to provide specific exercise guidelines or advice or refer cancer survivors to an exercise program than they were to recommend staying active. This is consistent with previous research [42, 43], and supports the rationale for a pathway addressing the challenge of connecting cancer survivors with services and resources by encouraging communication and collaboration between HCPs [16].

The top reported facilitators for promoting exercise were consistent with a survey of Canadian HCPs, who similarly nominated practitioner education sessions, educational handouts, and integration of an exercise specialist into the clinical team [32]. The two top reported barriers to discussing exercise, safety concerns and limited time, were also among the top four barriers reported by the Canadian sample [32]. Participants in our study additionally ranked poor knowledge about screening cancer survivors for their suitability to exercise and cancer survivors being told by others to rest among their top barriers. Collectively, these findings indicate multidisciplinary support for a team-oriented approach to exercise referrals and reinforce that cancer survivor, caregiver, and practitioner education is needed. Bone metastases were a commonly raised concern by participants who endorsed safety concerns as a barrier, supporting the recent development of evidence-based PA guidelines for individuals with bony metastases [15, 35]. Considerations and practical recommendations for addressing these and other barriers to implementing exercise counselling and referral in routine cancer care were recently proposed in a call-to-action detailing pathways for exercise programming, tailored to required individual levels of support and intervention [41]. Important implementation challenges to consider include under-resourced health systems without the infrastructure to facilitate assessment, triage, and referral of cancer survivors to appropriate exercise programming; lack of government subsidisation incentives; and gaps in workforce education and development opportunities [41]. Despite these potential barriers, there are actions that HCPs can take such as regularly assessing cancer survivors’ physical activity, providing cancer survivors with information about exercise guidelines, advising cancer survivors to be more active, and referring to supervised and unsupervised exercise programs as appropriate [41].

Alongside recall of guidelines, another predictor of participants’ current practice was whether they self-reported participating in leisure-time PA consistent with commonly recommended national standards. This is consistent with evidence indicating that HCPs who are physically active are more likely to provide PA counselling to their patients [37]. Further research is needed to clarify the nature of this relationship and determine whether promoting exercise among HCPs results in changes in cancer survivors’ exercise behaviours.

Strengths of this study included the use of a validated questionnaire based on the KTA framework [38, 39]. We expanded on previous studies by comparing the perspectives of different practitioner groups and examining predictors of their current practices. However, due to the sampling approach, it was not possible to determine a response rate or differences between participants and non-participants. Although we sought to recruit a diverse international sample, the survey was only available in English; most participants were recruited through North American, European, and Australian professional bodies affiliated with the study investigators; and the sample was weighted towards female participants from high-income countries. Given these limitations, the results can only serve as explorative in nature and may not be generalisable. Additionally, we did not collect data on the volume of cancer survivors seen by participants in their practice, which may have been a covariate. Finally, the impact of COVID-19 has limited many clinicians’ capacity to participate in research. This study did not set out to explore changes in practice during the pandemic; however, we are confident that the data describe HCPs’ general approach to exercise counselling, rather than changes during the pandemic.

HCPs play a fundamental role in addressing the relevance of exercise for cancer survivors, providing specific exercise advice, and making appropriate referrals in the context of personal, social, and environmental factors and resources [41]. Internationally, HCPs have reported gaps in knowledge and practical challenges regarding exercise counselling and referral for cancer survivors. Although participants routinely recommended PA to cancer survivors, additional education and practical support are needed to facilitate the provision of exercise guidelines, resources, and referrals. Our findings added to the understanding of individual factors associated with clinical practice, demonstrating that knowledge of exercise guidelines and weekly leisure-time PA were predictors of exercise counselling and referral. These results may inform strategies to enhance HCPs’ knowledge, interprofessional engagement, and access to local resources regarding exercise for cancer survivors.

## Supplementary Information

Below is the link to the electronic supplementary material.

Supplementary file1 (DOCX 14 KB)
