# Supplementary material for: Exercise counselling and referral in cancer care: an international scoping survey of health care practitioners’ knowledge, practices, barriers, and facilitators
Source: Support Care Cancer. 2022 Sep 29;30(11):9379–91. doi: 10.1007/s00520-022-07342-6 (PMC9521001; doi:10.1007/s00520-022-07342-6)
Supplement: Supplementary file 1 — Supplementary file1 (DOCX 14 KB) [file 520_2022_7342_MOESM1_ESM.docx]

Supplement 1: Oncology organisations and healthcare professional societies that promoted the study

| **Organisations and societies** |
| --- |
| Belgian Society of Radiation Oncology |
| Canadian Association of Nurses in Oncology |
| Cancer Nurses Society of Australia |
| Clinical Oncology Society of Australia |
| European Oncology Nursing Society |
| European Society for Radiotherapy and Oncology |
| Flemish Society for Nurses in Radiation Therapy and Oncology |
| Medical Oncology Group of Australia |
| Multinational Association for Supportive Care in Cancer |
| Psycho-oncology Co-operative Research Group |
